# Dietary Carbohydrate Promotes Cell Survival in Cancer Via the Up-Regulation of Fat Mass and Obesity-Associated Gene Expression Level

**DOI:** 10.21315/mjms2019.26.2.2

**Published:** 2019-04-30

**Authors:** Saeid Doaei, Maryam Gholamalizadeh, Mohammad Esmaeil Akbari, Shayan Akbari, Hyuliya Feradova, Ghazaleh Rahimzadeh, Alireza Mosavi jarrahi

**Affiliations:** 1 Student Research Committee, Cancer Research Center, Shahid Beheshti University of Medical Sciences, Tehran, Iran; 2 Gastrointestinal and Liver Disease Research Center, Guilan University of Medical Sciences, Rasht, Iran; 3 Department of Health Education, School of Health, Guilan University of Medical Sciences, Rasht, Iran; 4 Cancer Research Center, Shahid Beheshti University of Medical Sciences, Tehran, Iran; 5 Department of Nutrition, Iran University of Medical Sciences, Tehran, Iran; 6 Department of General Surgery, UMHAT St. Marina, Medical University of Pleven, Bulgaria; 7 Institute for Intelligent Systems Research and Innovation (IISRI), Deakin University, Geelong Waurn Ponds, Australia; 8 School of Medicine, Shahid Beheshti University of Medical Sciences, Tehran, Iran

**Keywords:** cancer, FTO gene, dietary carbohydrate

## Abstract

Cancer cells are mainly dependent on glycolysis for their growth and survival. Dietary carbohydrates play a critical role in the growth and proliferation of cancer and a low-carbohydrate diet may help slow down the growth of tumours. However, the exact mechanisms behind this effect are unclear. This review study aimed to investigate the effect of fat mass and obesity-associated (FTO) gene in the association between dietary carbohydrates and cancer. This study was carried out using keywords such as polymorphism and/or cancer and/or dietary carbohydrate and/or FTO gene. PubMed and Science Direct databases were used to collect all related articles published from 1990 to 2018.

Recent studies showed that the level of FTO gene expression in cancer cells is dramatically increased and may play a role in the growth of these cells through the regulation of the cellular metabolic pathways, including the phosphoinositide 3-kinases/protein kinaseB (PI3K/AKT) signaling pathway. Dietary carbohydrate may influence the FTO gene expression by eliminating the inhibitory effect of adenosine monophosphate-activated protein kinase (AMPK) on the FTO gene expression. This review summarised what has been recently discovered about the effects of dietary carbohydrate on cancer cells and tried to determine the mediating role of the FTO gene in these effects.

## Introduction

Recent studies suggested that healthy diet can play a major role in the prevention of cell malignancy, apoptosis of cancer cells and reduced tumour size (1–6). For example, the role of many vitamins and phytochemicals (such as curcumin) in cellular division and apoptosis has been frequently reported (7–9). More recently, the role of macronutrients such as carbohydrate in controlling the growth of cancer cells has attracted the attention of scientists (10). Cancer cells are dependent on their access to glucose for growth and reproduction; controlling blood glucose at an optimal level in cancer patients may be a practical way to avoid increasing the size of a tumour (11). One of the most recommended diets is the ketogenic diet that may be effective in reducing cancer cell growth by reducing the level of glucose availability (12).

Until recently not a lot has been found about the existing mechanisms by which dietary components affect the formation of cancer cells. The results of most studies suggest that some part of this effect may be due to the effects of dietary intake on the expression of some of the genes involved in the cell metabolism and division. The relation between gene variations and risk of cancer is well documented (13, 14). Cancer is a genetic disease caused by changes in the genes which have a role in the control of the growth, division and function of our cells (14). When tumour suppressor genes such as breast cancer genes 1 and 2 (BRCA1, BRCA2), and P53 carry a mutation, the cells can grow out of control and may lead to tumour formation (15). Recent studies have focused on the genes that are influenced by the environmental factors like dietary intake. It is assumed that the amount of nutrients available to the cell may modify the level of expression of some genes associated with cell growth and proliferation (16, 17). In other words, it is possible that dietary components play a major role in the risk of developing cancer through their effects on the expression of certain genes involved in the growth and proliferation of cancer cells (17). One of the genes that has recently been featured in this context is the fat mass and obesity-associated (FTO) gene (18). The association between the FTO gene and obesity has been confirmed through the study of the FTO gene polymorphisms. People who have the risk allele in the polymorphisms of the FTO gene (e.g. rs9939609 & rs9930506) have a greater risk of developing obesity (19). The amount of FTO gene expression in various tissues is also linked to body fat percentage and obesity (20). More recently, studies have shown that some of the effects of the FTO gene may be due to the impact of the intron regions of this gene on the expression of other genes, including the iroquois homeobox gene 3 (IRX3 gene) (21). The interactions between diet and the FTO gene have been recently reported in some studies (22). Considering the role of carbohydrates in cancer cell metabolism, this study aimed to investigate the mediating role of the FTO gene in the effect of dietary carbohydrates on cancer cells. First, we reviewed the role of several diets with different amounts of macronutrients administered to cancer patients. Then, we studied the role of the macronutrients in FTO gene expression. The probable molecular mechanisms of the association between the FTO gene and cancer development were also reviewed.

## Methodology

PubMed, PsycInfo and the Cochrane databases were searched to identify articles published in relevant fields. Appropriate keywords including carbohydrate, diet, FTO expression, FTO genotype, cancer, cell and metabolism (alone and together) were used to collect the papers. All articles published in English from June 1990 to July 2018 were studied. Of the total 180 articles, 109 articles were excluded because they failed to address the role of the FTO gene in breast cancer and/ or obesity and 63 articles for lack of sufficient information on the mechanism of the effects of FTO gene on the breast cancer and obesity. Finally, eight articles were included. Of these studies, five studies were on the relationship between dietary carbohydrate and cancer, and five were related to the molecular mechanisms of dietary carbohydrate on FTO gene.

## Dietary Carbohydrate and Cancer

Unlike normal cells, most malignant cells are dependent on the availability of sugar in the blood constantly for energy supply and to meet demands for their metabolism. These cells are unable to metabolise fatty acids and ketone bodies due to mitochondrial dysfunction. Previous studies reported the benefits of a low-carbohydrate diets on human body weight and general health (23). Ho et al. compared the effects of a low-carbohydrate diet compared to a western diet on tumour growth in mice (24). They first designed a low carbohydrate diet containing 8% carbohydrate (% of total calories), 23% fat and 69% protein versus a western diet (55% carbohydrate, 23% protein, 22% fat). The results showed that the growth of the cancer cells in low-carbohydrate and high-protein group was slower than their growth in a high-carbohydrate western diet. There were no differences between the weights of the cancer-bearing mice in the two groups. In addition, mice with a low-carbohydrate diet had lower levels of blood sugar, insulin and lactate. Only one rat on western diets could have a normal lifespan with cancer-related death, while more than 50% of the mice with a low-carbohydrate diet had a normal lifespan. It is also suggested that a low-carbohydrate diet possibly slows down the growth of cancer cells by reinforcing the inhibitory effects on the mechanistic target of rapamycin (mTOR) pathway. Taken together, the findings of this study revealed the ability of a low-carbohydrate diet to slow down the development of cancer without any impacts on weight.

Moulton et al. evaluated the effects of two different ratios of carbohydrate and protein in the early development of breast tissue carcinogenesis in an animal study (25). After tumour induction, the mice were exposed to either a low-protein and high-carbohydrate [(LPHC); 15% and 60% of energy, respectively] diet or a high-protein and moderate-carbohydrate [(HPMC); 35% and 40% of energy, respectively] diet for 10 weeks. The rate of the palpable tumours in the HPMC group compared to the LPHC group was lower and the level of insulin serum in the LPHC group was significantly higher than in the HPMC group. The researchers concluded that a low-carbohydrate diet can help to slow down the growth of breast tumours. The insulin-like growth factor 1 (IGF-1) system was reported as a key regulator of the cancer growth pathway and it seems that the reduction of insulin levels through diet or medication (e.g. metformin) was beneficial in cancer patients (26).

Sieri et al. conducted a cohort study and examined whether the glycemic load (GL) and the glycemic index (GI) are associated with the risk of breast cancer in women (27). A total of 289 patients with breast cancer were followed after 11.5 years. The results showed that the relative risk of the development of cancer with high GI and high GL were 1.57 and 2.53, respectively (*P* < 0.05). The risk of breast cancer was associated with the consumption of carbohydrates with higher GI_s_ (*P* for trend < 0.001).

The association between hyperglycemia and cancer risk was examined in 33,293 women and 31,304 men in northern Sweden (28). The results showed that the overall risk of cancer in women with the top quartile of fasting blood sugar (FBS) and blood sugar (BS) compared to bottom quartile was 1.75 versus 1.63. The risks of pancreatic, endometrium, urinary tract pipe and malignant melanoma cancers were associated with high FBS level, with the relative risk of 2.49, 1.86, 1.69, and 2.19, respectively.

Tan-Shalaby et al. examined the impact of the modified Atkins diet on cancer development. A total of 17 patients with advanced cancer, who had not undergone chemotherapy, were enrolled for the study (29). They received 20 g–40 g of carbohydrates daily for 16 weeks and were assessed every 4 weeks. The results showed that the modified Atkins diet is a safe and practical approach to prevent cancer progression and it can promote the quality of life. The survival time was also improved in some patients with lung cancer and melanoma.

Moreover, the effect of ketogenic diet as a well-known low-carbohydrate diet on tumour growth was investigated and approved in several studies (30–39). Reducing the amount of dietary carbohydrates is reported to suppress—or at least delay—the incidence of cancer and slow down the proliferation of cancer cells (30–34). Carbohydrates can have direct and indirect effects on the proliferation of cancer cells (40). A recent study identified that an increase in ketone bodies after a decrease in insulin and blood glucose had a negative effect on the proliferation of malignant cells in vitro during a calorie-restricted diet (41). It has recently been reported that the dependency of cancer cells on glycolysis (Warburg effect) may have a key role in the progression of cancer cells, but is not a cause of it (42). The studies on the mechanism of the effect of carbohydrates on the growth and proliferation of cancer cells reported that high levels of blood glucose and insulin will apply some of their effects through the steady activation of phosphoinositide 3-kinase/protein kinase B (PI3K/AKT) pathway that is involved in cell survival and eventually increase the risk of cancer (43, 44). However, the precise mechanism behind these effects of carbohydrate on this metabolic pathway is unclear.

## FTO Has a Possible Mediatory Role in the Association between Carbohydrates and Breast Cancer

FTO gene is known for its role in obesity and diabetes (19). The association between the FTO gene expression and cancer risk was also recently identified (45–47). The FTO gene may exert its effect through different mechanisms. It was reported that the level of FTO gene expression is higher in cancer cells than in normal cells and the cells adjacent to cancer tissue (45, 46). Moreover, the polymorphisms in the FTO gene are associated with cancer and may exert their role through their influence on the expression of the effective genes in cancer (47). However, some studies showed that there is no association between the FTO gene polymorphisms and the risk of cancer (48, 49). Yang et al. reported that there is no evidence that the gene variants in FTO are associated with a risk of colorectal cancer (49). In another study, the FTO rs11075995 variant risk allele was reported to be associated with breast cancer risk, without adjustment for body mass index (BMI). However, the adjustment to BMI led to the disappearance of this association, indicating that there is no independent association between the FTO polymorphism and the risk of breast cancer (50).

Recent studies on the relationship between the macronutrients intake and the level of FTO gene expression in the hypothalamus identified that dietary carbohydrates can affect the FTO gene expression level. However, the results in this area are contradictory and in some studies carbohydrate intake up-regulated the FTO gene expression, while in other studies it suppressed FTO gene expression (51). It is possible that this effects of dietary carbohydrates may depend on FTO genotype and FTO gene polymorphisms play a role in the association between carbohydrate intake and FTO gene expression (52, 53). Some recent studies showed the impact of carbohydrate intake on the association between FTO and BMI. A cross-sectional study on 4,839 Swedish people showed that the relation between FTO gene polymorphisms and obesity can be seen only in people who have a high rate of fat and low rate of carbohydrate intake (52). However, the results in this area were contradictory. For example, in a study by Qi et al. on 16,094 patients between 1 and 18 years, carbohydrate intake had no effect on the relationship between FTO and BMI (Table 1) (53).

**Figure 1:**
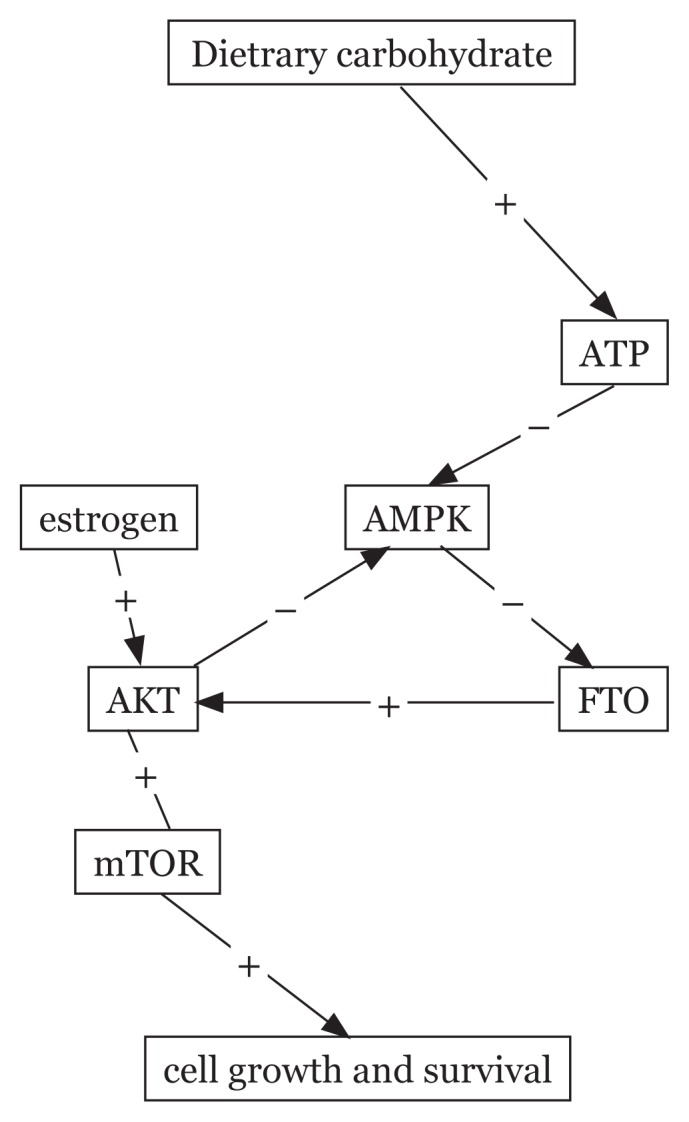
The effect of dietary carbohydrate on cell growth and survival through altering the FTO gene expression

The possible mechanism behind the effect of the FTO gene on cancer risk has been studied recently. The FTO gene may act as a mediator for the phosphorylation of serine in location 473 of the AKT protein and its activation has a key role in the proliferation and differentiation of cells (54). It was assumed that an increase in the FTO gene expression can increase glycolysis in the cancer cells by effect on the PI3K/AKT signaling pathway (55). It was also reported that the PI3K/AKT pathway is a mediator of the association between estrogen and the level of FTO gene expression and subsequent cell survival (56). It seems that the association between the FTO gene and cancer cells is more tangible in hormone-dependent cancers (Figure 1).

## Conclusion

Cancer cells are dependent on glycolysis for their growth and proliferation. Dietary carbohydrate is a significant factor and the association between blood glucose levels and cancer is well documented. But the exact mechanisms of this relationship remain unclear. Recent studies showed that dietary macronutrients may have an impact on cancer by altering the expression level of the genes associated with the metabolism of cancer cells (such as FTO). If we could improve the expression levels of the genes involved in growth, metabolism, and the function of cancer cells through changing our diet, we can hope to find a nutritionally applicable solution for the treatment and control of cancer in the future. Further research on the exact mechanism of the influence of FTO gene on the growth and proliferation of cancer cells may clarify the importance of this issue and the possibility of therapeutic use of dietary components in cancer.

## Figures and Tables

**Table 1:** Summary of study descriptions

Reference	Title	Study design	Examined components	Main finding(s)
Dietary carbohydrate and cancer				
Ho et al. (24)	A low-carbohydrate, high-protein diet slows tumour growth and prevents cancer initiation	Experimental	A low-carbohydrate, high-protein diet vs a high-carbohydrate, low-protein diet	The ability of a low-carbohydrate diet to slow down the development of cancer without any impact on weight
Moulton et al. (25)	A HPMC diet fed at discrete meals reduces early progression of N-methyl-N-nitrosourea-induced breast tumourigenesis in rats	Experimental	A low-protein and high-carbohydrate diet vs HPMC diet	A low-carbohydrate diet can help to slow down the growth of breast tumours
Sieri et al. (27)	Dietary GI, GL, and the risk of breast cancer in an Italian prospective cohort study	Cohort	High GI and GL vs low GI and GL	GL and GI are associated with the risk of breast cancer in women
Stattin et al. (28)	Prospective study of hyperglycemia and cancer risk	Cohort	FBS and BS of the top quartile versus the bottom quartile	Hyperglycemia was associated with cancer risk
Tan-Shalaby et al. (29)	KD in advanced cancer: A pilot feasibility and safety trial in the veterans affairs cancer patient population	CT	20 g–40 g of carbohydrates daily for 16 weeks	The modified Atkins diet is safe and a practical approach to prevent cancer progression and helps to maintain the quality of life
Shukla et al. (30)	Metabolic reprogramming induced by ketone bodies diminishes pancreatic cancer cachexia	Experimental	Normal diet vs KD	KD reduced tumour growth and inhibited body weight loss
Allen et al. (31)	KD enhance oxidative stress and radio-chemo-therapy responses in lung cancer xenografts	Experimental	Standard diet vs KD	KD enhances radio-chemotherapy responses in lung cancer xenografts by a mechanism that may involve increased oxidative stress
Kim et al. (32)	Carbohydrate restriction and in lactate transporter inhibition in a mouse xenograft model of human PCa	Experimental tumour	WD vs KD	Differences volumes were observed only in comparisons between mice fed a KD and mice fed a WD
Otto et al. (33)	Growth of human gastric cancer cells in nude mice is delayed by a KD supplemented with omega-3 fatty acids and MCT	Experimental	Standard diet vs KD	An unrestricted KD enriched with omega-3 fatty acids and MCT delayed tumour growth in a mouse xenograft model
Morscher et al. (34)	Inhibition of neuroblastoma tumour growth by KD and/or calorie restriction in a CD1-Nu mouse model	Experimental	Standard diet vs KD	KD reduced neuroblastoma tumour growth
Poff et al. (35)	The KD and hyperbaric oxygen therapy prolong survival in mice with systemic metastatic cancer	Experimental	Standard diet vs KD	KD produced anti-cancer effects in metastatic cancer
Caso et al. (36)	The effect of carbohydrate restriction on PCa tumour growth in a castrate mouse xenograft model	Experimental	WD vs KD	Carbohydrate restriction provided a benefit to slowing PCa tumour growth compared to a KD in mice
Abdelwahab et al. (37)	The KD is an effective adjuvant to radiation therapy for the treatment of malignant glioma	Experimental	Standard diet vs KD	KD significantly enhanced the anti-tumour effect of radiation
Caso et al. (36)	The effects of varying dietary carbohydrate and fat content on survival in a murine LNCaP prostate cancer xenograft model	Experimental	Moderate carbohydrate diet vs KD	Carbohydrate restriction improved the survival rate in PCa in humans
Hao et al. (39)	Growth of human colon cancer cells in nude mice is delayed by KD with or without omega-3 fatty acids and MCT	Experimental	Standard diet vs KD	An unrestricted KD delayed tumour growth in a mouse xenograft model
Dietary carbohydrates and the FTO gene				
Gholamalizadeh et al. (51)	Macronutrients and the FTO gene expression in hypothalamus; a systematic review of experimental studies	Systematic review of experimental studies	The effect of dietary carbohydrates on the FTO gene expression	Dietary carbohydrates can affect the level of FTO gene expression
Sonestedt et al. (52)	Fat and carbohydrate intake modify the association between genetic variation in the FTO genotype and obesity	Cross-sectional	The impact of carbohydrate intake on the association between FTO and BMI	The relation between FTO gene polymorphisms and obesity can be seen only in people who have a low carbohydrate intake
Qi et al. (53)	FTO genetic variants, dietary intake, and BMI: Insights from 177,330 individuals.	Cross-sectional	The impact of carbohydrate intake on the association between FTO and BMI	Carbohydrate intake had no effect on the relationship between FTO and BMI

Body mass index (BMI), glycemic index (GI), glycemic load (GL), fasting blood sugar (FBS), blood sugar (BS), fat mass and obesity-associated (FTO), clinical trial (CT), ketogenic diet (KD), western diet (WD), medium-chain triglycerides (MCT), prostate cancer (PCa), high-protein and moderate-carbohydrate (HPMC)
